# Three Gorges Dam: Potential differential drivers and trend in the spatio-temporal evolution of the change in snail density based on a Bayesian spatial–temporal model and 5-year longitudinal study

**DOI:** 10.1186/s13071-023-05846-6

**Published:** 2023-07-14

**Authors:** Yanfeng Gong, Yixin Tong, Honglin Jiang, Ning Xu, Jiangfan Yin, Jiamin Wang, Junhui Huang, Yue Chen, Qingwu Jiang, Shizhu Li, Yibiao Zhou

**Affiliations:** 1 Fudan University School of Public Health, Building 8, 130 Dong’an Road, Xuhui District, Shanghai, 200032 China; 2 Key Laboratory of Public Health Safety, Ministry of Education, Fudan University, Building 8, 130 Dong’an Road, Xuhui District, Shanghai, 200032 China; 3 Fudan University Center for Tropical Disease Research, Building 8, 130 Dong’an Road, Xuhui District, Shanghai, 200032 China; 4 School of Epidemiology and Public Health, Faculty of Medicine, University of Ottawa, 600 Peter Morand Crescent, Ottawa, ON K1G 5Z3 Canada; 5 Chinese Center for Disease Control and Prevention, NHC Key Laboratory of Parasite and Vector Biology, National Institute of Parasitic Diseases, Chinese Center for Tropical Diseases Research, Shanghai, 200025 China

**Keywords:** Three Gorges Dam, Snail abundance, Spatial–temporal effects, *Oncomelania hupensis* snail

## Abstract

**Background:**

Snail abundance varies spatially and temporally. Few studies have elucidated the different effects of the determinants affecting snail density between upstream and downstream areas of the Three Gorges Dam (TGD). We therefore investigated the differential drivers of changes in snail density in these areas, as well as the spatial–temporal effects of these changes.

**Methods:**

A snail survey was conducted at 200 sites over a 5-year period to monitor dynamic changes in snail abundance within the Yangtze River basin. Data on corresponding variables that might affect snail abundance, such as meteorology, vegetation, terrain and economy, were collected from multiple data sources. A Bayesian spatial–temporal modeling framework was constructed to explore the differential determinants driving the change in snail density and the spatial–temporal effects of the change.

**Results:**

Volatility in snail density was unambiguously detected in the downstream area of the TGD, while a small increment in volatility was detected in the upstream area. Regarding the downstream area of the TGD, snail density was positively associated with the average minimum temperature in January of the same year, the annual Normalized Difference Vegetation Index (NDVI) of the previous year and the second, third and fourth quartile, respectively, of average annual relative humidity of the previous year. Snail density was negatively associated with the average maximum temperature in July of the previous year and annual nighttime light of the previous year. An approximately inverted “U” curve of relative risk was detected among sites with a greater average annual ground surface temperature in the previous year. Regarding the upstream area, snail density was positively associated with NDVI and with the second, third and fourth quartile, respectively, of total precipitation of the previous year. Snail density was negatively associated with slope.

**Conclusions:**

This study demonstrated a rebound in snail density between 2015 and 2019. In particular, temperature, humidity, vegetation and human activity were the main drivers affecting snail abundance in the downstream area of the TGD, while precipitation, slope and vegetation were the main drivers affecting snail abundance in the upstream area. These findings can assist authorities to develop and perform more precise strategies for surveys and control of snail populations.

**Graphical Abstract:**

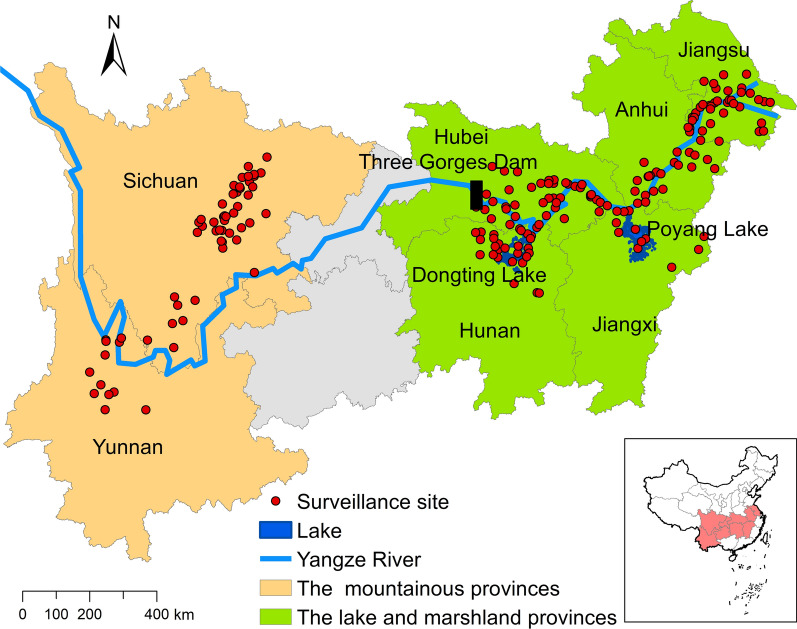

**Supplementary Information:**

The online version contains supplementary material available at 10.1186/s13071-023-05846-6.

## Background

The key to controlling schistosomiasis lies in disrupting the parasite's complex life-cycle [1]. The snail *Oncomelania hupensis* is the sole intermediate host of *Schistosoma japonicum *and therefore an indispensable link in the transmission of the disease [2]. There is a close association between the distribution of *O. hupensis* and schistosomiasis endemic areas [3, 4]. Snail control has been an effective strategy for the eradication of schistosomiasis [3, 4]. However, it has been estimated that the area covered by snail-infested settings in China remained at 3.6 billion m^2^ between 2012 and 2021 [5], with a trend toward enlargement in newly emerging and reemerging areas of habitats [6, 7].

The Three Gorges Dam (TGD) began filling with water in 2003. Considerable changes have been observed in water level, weed and silt sediment after the TGD became operational, which further impacted the ecological environment of snail survival [8–11]. The catastrophic floods in 2016 caused the spread of snails into a large area [6]. The "Outline of the Development Plan for the Yangtze River Economic Belt" released in 2016 emphasized ecological priority and green development, placing restrictions on the massive molluscicide [12]. All of these factors can influence the life history of snails but, in particular, the many impacts of dams on snails are associated with hysteresis and unpredictability [8–11]. Therefore, it is important to clarify the secular variation trend in snail abundance for early identification of potential risks of schistosomiasis transmission. Few studies have focused on the long-term spatial–temporal evolution of snail density associated with the TGD.

Many studies have shown that environmental factors such as climate and vegetation are important factors affecting *O. hupensis* [13, 14]. Zhang et al. [15] showed that the number of snails was negatively correlated with soil temperature and vegetation height, and positively correlated with soil moisture. Wu et al. [16] reported that the suitable ranges of water content, pH, soil temperature and altitude of snails in Dongting Lake were 58.7–68.9%, 6.6–7.0, 22.73–24.23 °C and 23.5–26.0 m, respectively. Snail density varies spatially and temporally in different geographical regions. However, to date, no large-scale longitudinal studies have been conducted to determine the different impacts of environmental factors on snail density between the upstream (hilly) and the downstream (mainly lake and marshland) areas of the TGD where approximately 96.8% of all snail habitats in mainland China are located [17].

Human activities are also related to snail distribution. Olkeba et al. [18] showed that anthropogenic activities, such as human settlement, drainage of land and silviculture, are important determinants for the occurrence and abundance of snail species. Night light and human footprints play an important role in risk prediction of infectious diseases and their vectors [19]. Night light may reflect human activities as a determinant of snail density.

Bayesian inference is a useful method for data modeling that estimates posterior distribution of model parameters by updating prior distributions with information provided by recorded observations [20]. The Bayesian spatio-temporal model can take into account spatial autocorrelation and temporal dependence when analyzing covariates, which makes it a more flexible approach for modeling a small number of spatially correlated data [21]. In the present study, we used a Bayesian spatio-temporal model to explore the relationships of snail density with environmental and social factors, as well as the spatial–temporal effects on the change of snail density. The aim of this study was to elucidate possible differential drivers of the change in snail density between the downstream and upstream areas of the TGD, as well as the trend in the spatio-temporal evolution of snail density between the 12th and 17th year after the TGD became operational.

## Methods

### Study area

**Fig. 1:**
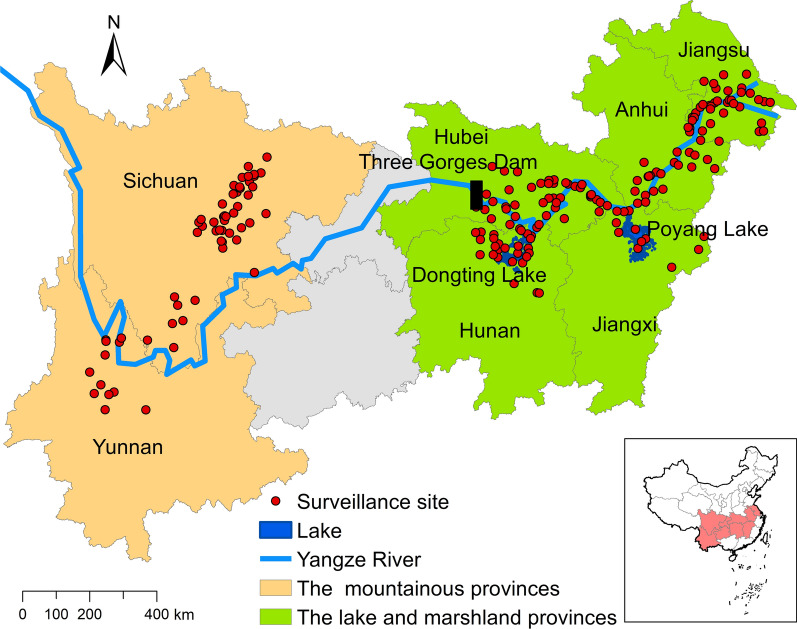
Schistosomiasis endemic areas and distribution of monitoring sites in the Yangtze River basin

The Yangtze River basin is located in the south of China. The region has an annual average temperature of between 16 °C and 18 °C and an average annual precipitation of 1067 mm. The seven provinces in this area where schistosomiasis is endemic and which have not yet met the elimination standard can be categorized into the hilly endemic area in the upstream area of the TGD (Sichuan and Yunnan) and the endemic area dominated by lakes and marshland in the downstream area of the TGD (Hubei, Hunan, Jiangxi, Anhui and Jiangsu) (Fig. 1).

### Snail data

According to the National Schistosomiasis Surveillance Program (2014 edition), village-based monitoring sites were set up in schistosomiasis-endemic counties throughout the country [22]. A snail survey was conducted annually in the spring between 2015 and 2019 in areas infested with snails or suspected of being infested with snails. The method involved using a square frame made of iron wire (0.1 m^2^), in a systematic sampling method paired with an environmental sampling method [23]. For susceptible environments, a systematic sampling method was applied to investigate snails (the distance between the borders of rivers, lakes, islands and beaches was 20 m, and the distance between borders in other environments was 10 m) [22]. For environments where the presence of snails was suspected, a systematic sampling method combined with an environmental sampling method was conducted [22].

The longitude and latitude of each environment were recorded using a handheld global positioning system (GPS) device [24]. The collected snails were identified as dead or alive using the knocking method, and the infection status of live snails with *S. japonicum* was detected using under the dissecting microscope [24]. At the same time, loop-mediated isothermal amplification (LAMP) was used to detect the nucleic acid of *S. japonicum* in the snails [24]. Data on the geographical location, environmental name, number of living snails in the system sampling and number of survey frames in the system sampling in the Yangtze River basin were collected, sorted and verified. A total of 200 monitoring sites were included, of which 142 were in the downstream area and 58 were in the upstream area (Fig. 1).

### Environmental data

Average annual temperature (Tem, °C), average annual relative humidity (RH, 1%), annual number of hours of sunshine (SSH) and average annual ground surface temperature (GST, °C) were obtained from the National Meteorological Science Data Center (https://data.cma.cn/). Grid data of these four factors were obtained using the ordinary kriging method (gstata package in R) based on the observational station data. Data on the average minimum temperature in January (Tmin, °C), average maximum temperature in July (Tmax, °C) and total precipitation (Pre, mm) were obtained from the National Earth System Science Data Center (http://www.geodata.cn/). Distance to major waterway (DW, km) and slope (SLP) were obtained from the Open Spatial Demographic Data and Research Repository (https://hub.worldpop.org/). The annual Normalized Difference Vegetation Index (NDVI) and annual nighttime light (NL) were obtained from the Resource and Environmental Science and Data Center (https://www.resdc.cn/). The data on monitoring sites were extracted using the raster package in R.

*Oncomelania hupensis* snails typically lay eggs in the spring (between March and May). It usually takes about 1 month at a suitable temperature for the egg to hatch, then the young snail grows and develops into the adult snail. The life span of snails is about 1 year. Therefore, the snails detected in the spring are mainly affected by the environmental factors of the previous year, with the exceptions that snails are affected by the Tmin of the same year. The SLP and DW of surveillance sites changed little during the study period. The relationship between time period and snail density and associated variables is shown in Table 1.

**Table 1:** Relationship between time period and snail density and associated variables

Variables (abbreviation, unit)	Time period
Snail density in the spring of the year (snail density, /0.1 m^2^)	2015–2019
Average annual temperature of the previous year (Tem, °C)	2014–2018
Average minimum temperature in Jan of the same year (Tmin, °C)	2015–2019
Average maximum temperature in Jul of the previous year (Tmax, °C)	2014–2018
Total precipitation of the previous year (Pre, mm)	2014–2018
Average annual relative humidity of the previous year (RH, 1%)	2014–2018
Annual sunshine hours of the previous year (SSH, h)	2014–2018
Average annual ground surface temperature of the previous year (GST, °C)	2014–2018
Distance to major waterway (DW, km)	—
Normalized Difference Vegetation Index of the previous year (NDVI)	2014–2108
Slope (SLP)	—
Annual nighttime light index of the previous year (NL)	2014–2018

### Data preprocessing

The living snail density for each surveillance site was calculated by dividing the number of live snails by the number of survey frames in the system sampling. The expected number of snails was calculated by multiplying the number of survey frames by the average snail density. Spearman’s rank correlation coefficient and variance inflation factor (VIF) were calculated between variables. A VIF value > 10 indicates that there is strong collinearity between variables and these cannot be included in the model.

### Bayesian spatial–temporal model

A Bayesian spatial–temporal model was used to analyze the spatio-temporal and environmental and social effects through the inclusion of adjacent spatio-temporal information. The general form of the model is given as follows [25, 26]:

$${y}_{it}\sim NB\left({\pi }_{it},\mathrm{r}\right)$$

$${\pi }_{it}=r/(r+{u}_{it})$$

$$log\left({u}_{it}\right)=log{e}_{it}+\sum_{k=1}^{k}{\beta }_{k}{X}_{itk}+{u}_{i}+{v}_{i}+{\varphi }_{t}+{\gamma }_{t}+{\delta }_{it}$$

where ${y}_{it}$ denotes the actual number of live snails, ${e}_{it}$ denotes the expected number of snails in $i$ site for $t$ year. The model assumes that ${y}_{it}$ is distributed according to a negative binominal distribution, with a mean ${u}_{it}={e}_{it}{\theta }_{it}$, where ${\theta }_{it}$ represents the relative risk (RR) in $i$ site for $t$ year.* r* denotes the dispersion indication. ${\beta }_{k}$ represents the vector of the regression parameters; ${X}_{itk}$ represents the* k*th variable in $i$ site for $t$ year. ${v}_{i}$ and ${\varphi }_{t}$ represent the spatially unstructured effect and temporally structured effect, respectively, both of which follow a normal distribution. ${u}_{i}$ represents the spatially structured effect that follows a conditional autoregressive process (CAR). The CAR adjacent matrix $\mathrm{w}$ is defined by K nearest neighbor. ${\gamma }_{t}$ represents the temporally structured component using the first-order autoregression. ${\delta }_{it}$ denotes the spatial–temporal interaction effect that follows a normal distribution.

We attempted to sequentially add time, space and space–time interaction effects on the basis of the Bayesian non-spatio-temporal model, and five Bayesian models were constructed (Additional file 1: Table S1). The five models were compared by the deviance information criterion (DIC), which comprehensively considers the fitting and complexity [27], and then the best model was selected to analyze the spatial–temporal effects on the change in snail density, as well as environmental and social factors related to snail density. The RR associated with temporal, spatial, spatio-temporal interaction and environmental effects were calculated by $exp\left({\varphi }_{t}+{\gamma }_{t}\right)$, $exp\left({u}_{i}+{v}_{i}\right)$, $exp\left({\delta }_{it}\right)$ and $exp\left({\beta }_{k}\right)$, respectively. The RR for the spatial effect was divided into three types according to Richardson's classification rules [28] as follows: hot spot ($p[exp\left({u}_{i}+{v}_{i}\right)>1]\ge 0.8$), cold spot ($p[exp\left({u}_{i}+{v}_{i}\right)>1]<0.2$) and other spots ($0.2\le p[exp\left({u}_{i}+{v}_{i}\right)>1)]<0.8$). Models were conducted using integrated nested Laplace approximation methods in the R-INLA package in R.

## Results

### Characteristics of snail density and environmental variable

**Fig. 2:**
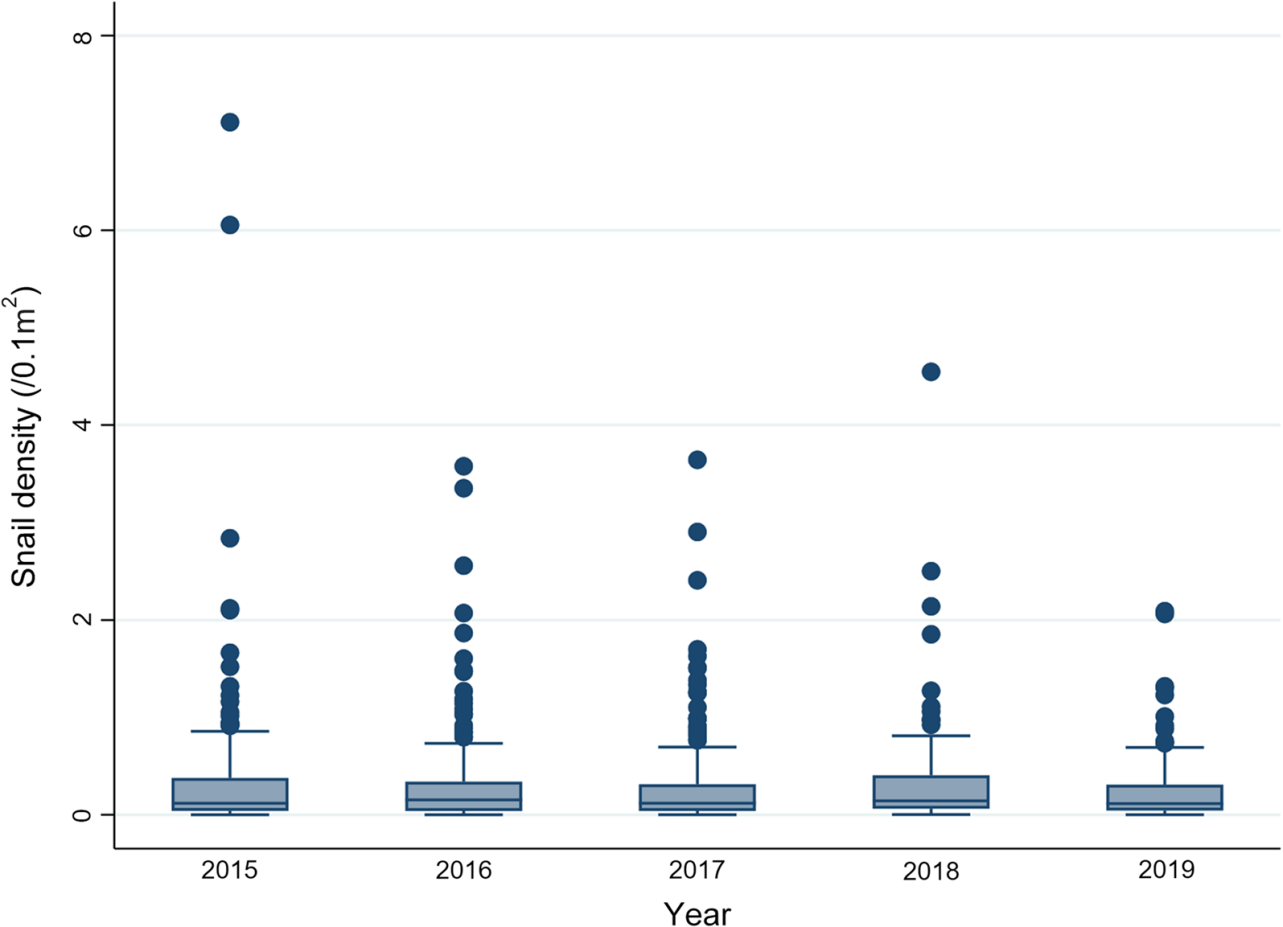
Boxplot of snail densities in the Yangtze River basin between 2015 and 2019

Median snail density in the Yangtze River basin from 2015 to 2019 was 0.118, 0.153, 0.118, 0.143 and 0.114 snails/0.1 m^2^, respectively. The number of sites with extremely high snail density decreased over the study period (Fig. 2).

A description of the climate and geographical conditions is shown in Table 2. Median Tem, Tmin, Tmax, Pre, RH, SSH, GST, DW, NDVI, SLP and NL were 17.073 °C, 2.200 °C, 33.100 °C, 1304.000 mm, 77.6%, 6233 h, 19.400 °C, 1.540 km, 0.719, 1.000 and 0.568, respectively (Table 2).

**Table 2:** Descriptive statistics of climate and geographical variable

Variables	Mean	SD	Minimum	P_25_	Median	P_75_	Maximum
Tem (°C)	16.847	1.393	8.853	16.383	17.073	17.720	19.261
Tmin (°C)	2.223	1.736	− 2.100	1.100	2.200	3.300	8.900
Tmax (°C)	32.260	2.547	24.500	30.880	33.100	34.300	36.200
Pre (mm)	1364.000	379.531	554.000	1102.000	1304.000	1590.000	2625.000
RH (%)	79.3	4.9	52.2	75.1	77.6	79.3	85.9
SSH (h)	6153.000	509.136	3231.000	5980.000	6233.000	6472.000	7037.000
GST (°C)	19.130	1.302	12.170	18.530	19.400	19.900	22.820
DW (km)	2.382	2.621	0.000	0.548	1.540	3.341	14.386
NDVI	0.674	0.167	0.000	0.580	0.719	0.796	0.900
SLP	2.915	4.766	0.000	0.000	1.000	2.250	27.000
NL	3.299	6.858	0.007	0.299	0.568	2.566	42.896

*SD* standard deviation, *P*_*25*_ 25th percentile, P_*75*_ 75th percentile,* Tem* annual average temperature, *Tmin* average minimum temperature in January, *Tmax* average maximum temperature in July, *Pre* total precipitation, *RH* average annual relative humidity, *SSH* number of sunshine hours, *GST* average annual ground surface temperature, *DW* distance to major waterway, *NDVI* normalized difference vegetation index, *SLP* slope, *NL* annual nighttime light index

### Variable inclusion and model selection

The Spearman correlation coefficients among Tem, SSH and GST were > 0.90 (*P* < 0.05), all other Spearman correlation coefficients were < 0.50 (Additional file 1: Table S2). The VIF values of Tem and SSH were much greater than 10, indicating that there was serious collinearity (Additional file 1: Table S3). Based on the above results, Tem and SSH were excluded from the models. Because there may be nonlinear relationships between snail density and rainfall, humidity or temperature, this study divided Pre, RH and GST into four quartiles (Q1, Q2, Q3, Q4) for Bayesian modeling. The DIC of model 5, which included spatial, temporal and spatio-temporal interaction terms, was less than the DIC values of the other four models (Additional file 1: Table S4). Model 5 (Bayesian spatio-temporal interaction model) was selected to explore the determinants of and spatial–temporal effects on the change in snail density.

### Determinants associated with change in snail density

For the downstream areas (Table 3), Tmin was positively associated with increased snail density (RR 1.117, 95% confidence interval [CI] 1.013–1.232), while Tmax was negatively associated with increased snail density (RR 0.980, 95% CI 0.968–0.993). NDVI was positively related to increased snail density increment (RR 1.164, 95% CI 1.141–1.188), and NL was negatively correlated with increased snail density (RR 0.886, 95% CI 0.862–0.910). An increasing trend was detected among sites with a greater RH, compared with sites in the first quartile cluster; sites in the second, third and fourth quartile clusters had higher RR values for snail density, which were 1.157 (95% CI 1.146–1.169), 1.719 (95% CI 1.702–1.735), 1.487 (95% CI 1.480–1.494), respectively. An inverted "U" curve of "increase first and then decrease" was detected among sites with a greater GST, compared with sites in the first quartile cluster; the RR values of sites in the second, third and fourth quartile clusters were 1.017 (95% CI 1.010–1.023), 1.208 (95% CI 1.201–1.216) and 0.673 (95% CI 0.667–0.678), respectively.

**Table 3:** Posterior estimates of relative risk for factors related to snail density

Related factors	RR1 (95% CI)^a^	RR2 (95% CI)^a^
Tmin	1.117 (1.013–1.232)*	0.919 (0.804–1.049)
Tmax	0.980 (0.968–0.993)*	0.991 (0.964–1.019)
Q1 Pre (lowest)^b^	1(Ref)	1(Ref)
Q2 Pre	0.860 (0.678–1.091)	1.076 (1.068–1.084)*
Q3 Pre	0.871 (0.666–1.138)	1.339 (1.329–1.349)*
Q4 Pre (highest)	0.831 (0.614–1.126)	1.109 (1.100–1.119)*
Q1 RH (lowest)^b^	1(Ref)	1(Ref)
Q2 RH	1.157 (1.146–1.169)*	1.050 (0.803–1.374)
Q3 RH	1.719 (1.702–1.735)*	1.015 (0.758–1.359)
Q4 RH (highest)	1.487 (1.480–1.494)*	1.141 (0.837–1.555)
Q1 GST (lowest)^b^	1(Ref)	1(Ref)
Q2 GST	1.017 (1.010–1.023)*	0.989 (0.743–1.316)
Q3 GST	1.208 (1.201–1.216)*	0.900 (0.668–1.213)
Q4 GST (highest)	0.673 (0.667–0.678)*	0.923 (0.655–1.299)
DW	0.986 (0.904–1.077)	1.127 (0.989–1.285)
NDVI	1.164 (1.141–1.188)*	1.189 (1.160–1.219)*
SLP	0.975 (0.930–1.024)	0.910 (0.865–0.958)*
NL	0.886 (0.862–0.910)*	0.945 (0.891–1.001)

*RR* relative risk, *CI* confidence interval, *Tmin* average minimum temperature in Jan, *Tmax* average maximum temperature in Jul, *Pre* total precipitation, *RH* average annual relative humidity, *GST* average annual ground surface temperature, *DW* distance to major waterway, *NDVI* normalized difference vegetation index, *SLP* slope, *NL* annual night-time light index

^*^ The increased snail density was significantly different at *P* < 0.05, Q1 Pre, Q1 RH and Q1 GST were the reference group in Pre, RH, and GST, respectively

^a^RR1 and RR2 represent the relative risk of increased snail density in the downstream and the upstream areas of the Three Gorges Dam, respectively

For the upstream area (Table 3), the NDVI was significantly and positively associated with increased snail density (RR 1.189, 95% CI 1.160–1.219), and SLP was negatively associated with increased snail density (RR 0.910, 95% CI 0.865–0.958). An increasing trend was detected among sites with a greater total Pre, compared with sites in the first quartile cluster; the RR values of sites in the second, third and fourth quartile clusters were 1.076 (95% CI 1.068–1.084), 1.339 (95% CI 1.329–1.349) and 1.109 (95% CI 1.100–1.119), respectively.

### Spatial–temporal effects on the change in snail density

**Fig. 3:**
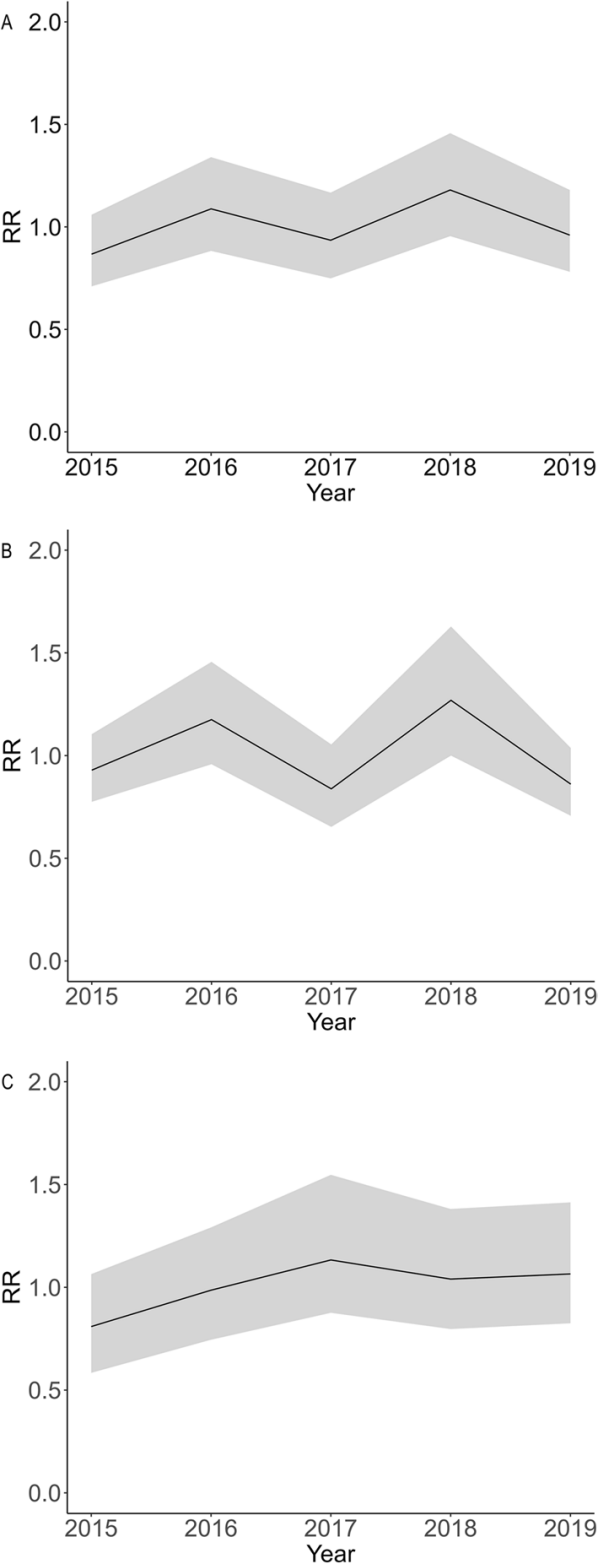
Temporal effects of RR for the increased snail density from 2015 to 2019 in the Yangtze River basin (**a**), the downstream area of the Three Gorges Dam (**b**) and the upstream area of the Three Gorges Dam (**c**). RR, Relative risk

A fluctuating and small upward trend for snail density was detected in the Yangtze River basin from 2015 (RR 0.867, 95% CI 0.710–1.059) to 2019 (RR 0.960, 95% CI 0.781–1.180). Separately, an obvious volatility trend was observed in the downstream area, while a small increasing trend was detected in the upstream area (Fig. 3).

**Fig. 4:**
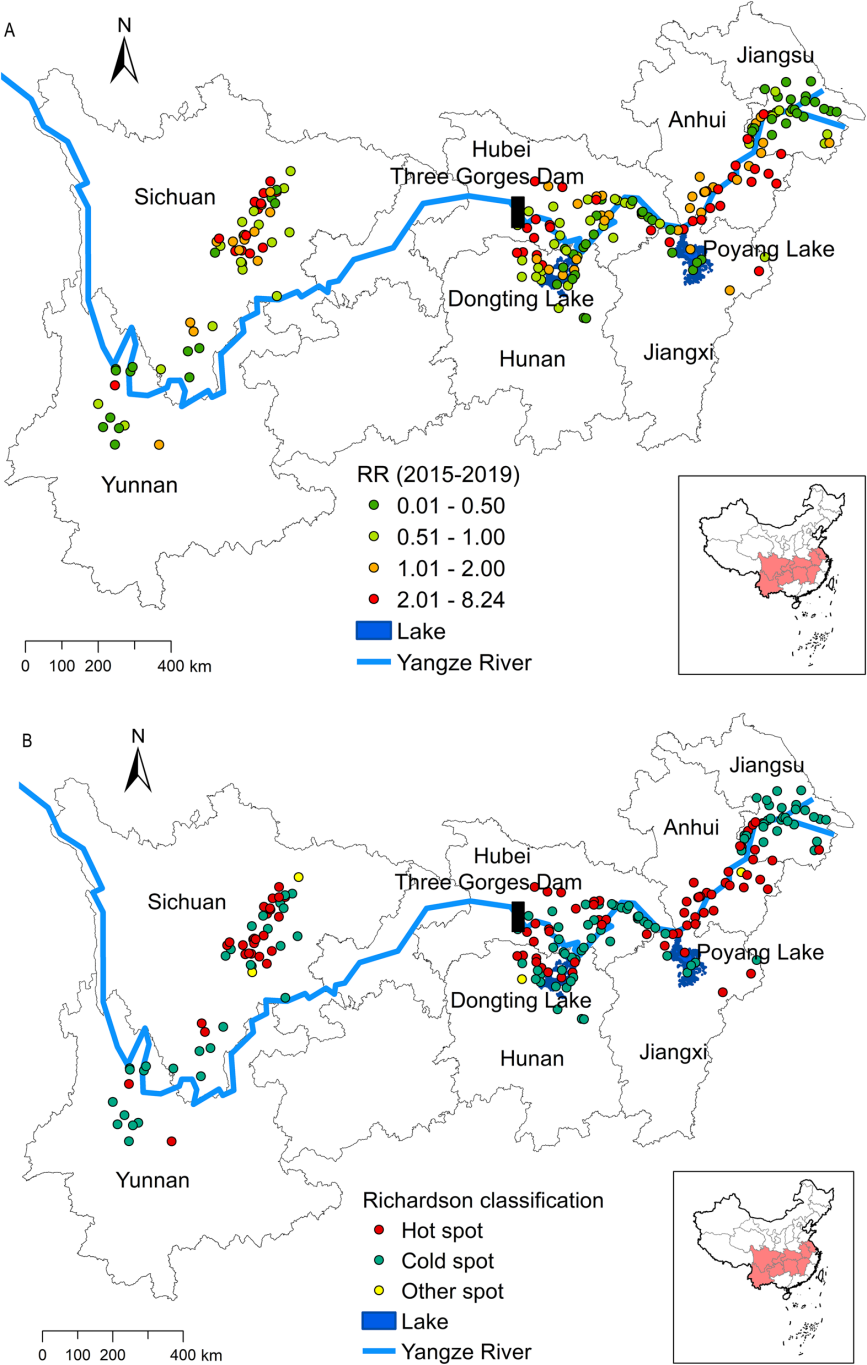
Spatial effects (**a**) and cold and hot spots (**b**) of the RR for the increased snail density. RR, Relative risk

A spatially obvious difference of RR for the increasing trend in snail density was detected in the Yangtze River basin (Fig. 4). The estimated RR $[exp\left({u}_{i}+{v}_{i}\right)]$ for the increase in snail density was relatively high in the northwest of Dongting Lake, the Jianghan Plain, the north of Poyang Lake, the Anhui section of the Yangtze River and the Chengdu Plain. According to Richardson's classification rules, there were 89 (44.5%) hot spots, five (2.5%) stable spots and 106 (53.0%) cold spots (Fig. 4b). Among all hotspots, 63 (70.8%) hotspots were located in the downstream area.

**Fig. 5:**
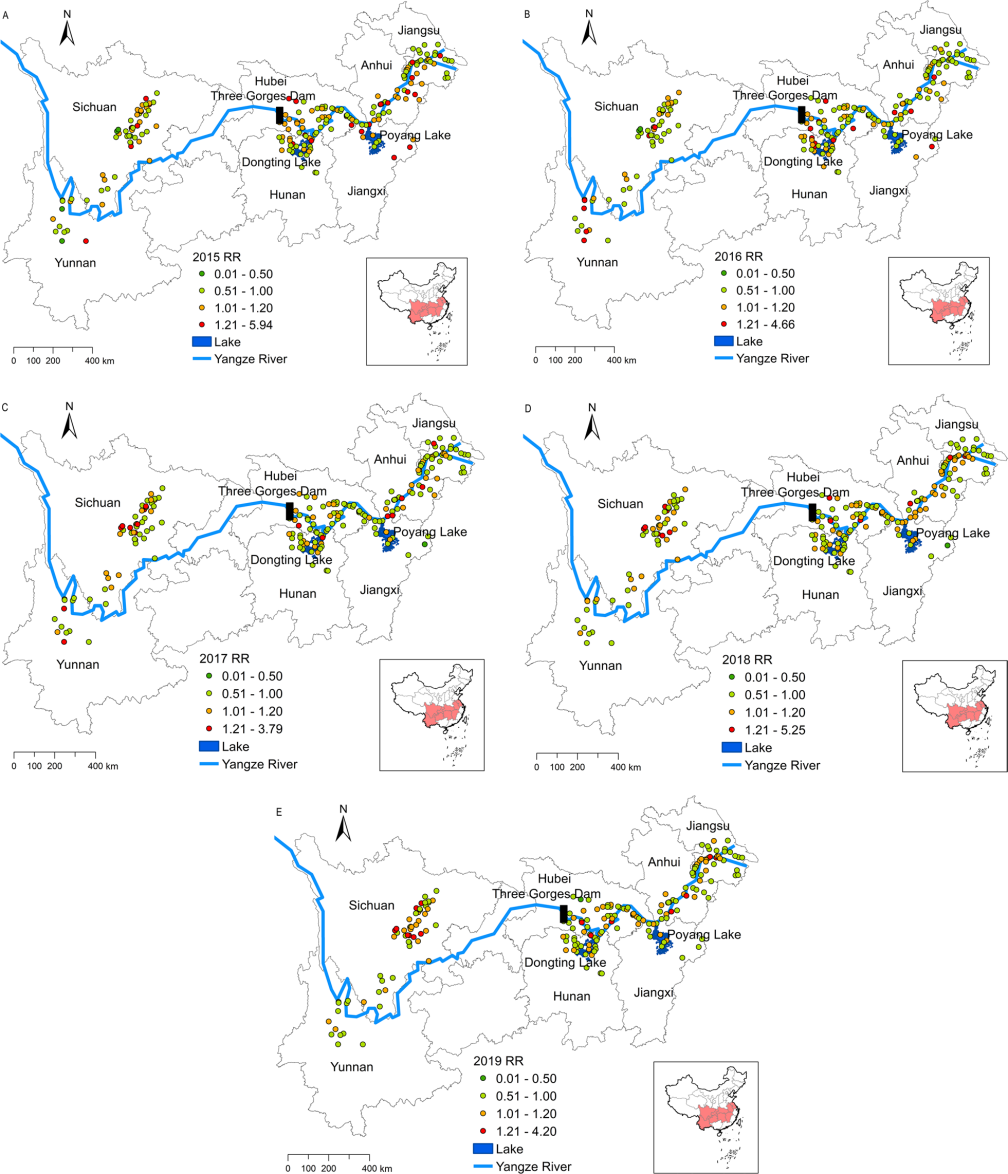
Spatial–temporal interaction effect of the RR for the increased snail density. **a**–**e** Results from 2015 (**a**), 2016 (**b**), 2017 (**c**), 2018 (**d**), 2019 (**e**). RR, Relative risk

The results of the study on spatial–temporal interaction effects are mapped in Fig. 5. A fluctuation trend of estimated RR values for the increase in snail density from 2015 to 2019 was detected in the Donting Lake, Poyang Lake, Jianghan Plain and Anhui section of the Yangtze River, whereas an increasing trend was observed in the Chengdu Plain. From 2015 to 2019, the number of sites with a RR value > 1 for the increased snail density was 90 (2015), 70 (2016), 65 (2017), 86 (2018) and 81 (2019), with a trend of "falling first and then rising."

## Discussion

### Drivers associated with the change in snail density

After adjusting for spatial–temporal effects, the Bayesian models showed that Tmin was positively associated with snail density (RR 1.117, 95% CI 1.013–1.232) in the downstream area and that Tmax was negatively associated with snail density (RR 0.980, 95% CI 0.968–0.993). The *O. hupensis* snail is a narrow-temperature and amphibious freshwater snail, and extreme weather affects the abundance of snail populations [29]. Influence model simulations showed that *O. hupensis* snails in the middle and lower reaches of the Yangtze River may theoretically move northward, especially following implementation of the South-to-North Water Transfer Project; however, to date snail habitats north of the “frost line” have not been detected [30–33]. The sensitivity of snails to extreme temperatures may be the key factor limiting their northward spread. Previous experiments showed that while the development rate of snail eggs accelerated with increasing temperature, the development rate was inhibited at higher or lower temperatures [34, 35]. This is basically consistent with the findings of the current study (referenced against Q1 GST, snail density was positively associated with the Q2 GST and Q3 GST, but negatively associated with Q4 GST; Table 3) ).

Humidity and vegetation are also closely related to snail breeding. Our study demonstrated that NDVI was positively related to snail density (RR 1.164, 95% CI 1.141–1.188), and an increasing trend in snail density was detected among sites with a greater RH. Snails lay most eggs in semi-humid soil and do not lay eggs in dry surroundings [36]. Weeds, reeds and other vegetation play an essential role in keeping the soil moist, and regulating temperature and shading in the breeding habitats [36]. Noteworthy, this study revealed that NL was negatively correlated with snail density (RR 0.886, 95% CI 0.862–0.910). Nighttime light is an indirect indicator reflecting human activities and socioeconomic conditions and is related to snail density through the environment [37]. Sites with lower nighttime light usually denote good ecological conditions and lower human impact on the environment [37]; hence, the environment with lower nighttime light is conducive to the breeding and reproduction of snails.

In the upstream snail-infested sites of the TGD, a significantly increasing trend in snail density was detected with a greater Pre, and snail density was negatively associated with SLP (RR 0.910, 95% CI 0.865–0.958). This may be caused by the unique geography of the upstream area [38]. The upstream snail-infested sites are found in the hilly and mountainous habitats where snails are mostly distributed in weedy rivers, ditches and other environments along mountain streams [39]. Precipitation is an important factor that supplements surface runoff in hilly areas [40]. Adequate rainfall, small slope and lush vegetation are conducive to the survival and settlement of snails in hilly areas. Snail-infested sites in the upstream area are commonly characterized by lower economic development and less human interference (regardless of the high or low snail density) [38, 40], which may explain why this study does not find an statistical correlation between snail density and night light in the upstream area.

### Spatial–temporal effects on the change of snail density

The number of sites with extremely high snail density was found to be decreasing, indicating that snail control measures have had a certain amount of success. However, in contrast to previous studies showing that snail density trended downwards between 2003 and 2015 after the TGD became operational [9, 10], we detected an obvious fluctuating and small increasing trend for snail density between 2015 and 2019 (Fig. 3). The volatility of snail density may be associated with frequent flooding disasters that are considered to be responsible for the spread of snails and the variation in snail density [41]. The implementation of massive molluscicide and environmental modification projects became unrealistic, affected by the rigorous policy of the Yangtze River Protection and Ecological Restoration Project [24, 42]. Under the dual influence of natural and social factors, the snail density showed an obvious fluctuation and a slightly increasing trend during the study period.

In terms of spatial effect, this study demonstrated that the high-risk areas for increased snail density were northwest of Dongting Lake, the Jianghan Plain, north of Poyang Lake, the Anhui section of the Yangtze River and the Chengdu Plain. Among all hotspots, 70.8% (63/89) were located in the downstream area of the TGD (Fig. 4). These results are in accordance with suitability prediction based on ecological niche model [17, 43]. Control of the water level is extremely difficult along beaches in the downstream area of the TGD [44]. The characteristics of “winter land and summer water” are conducive to the growth and reproduction of amphibious snails [9]. Once functional, the TGD lowered the water level in the downstream area and compressed the breeding space of snails in high-elevation bottomlands [11, 45]. However, it has also been observed that the extension of the dry season and the normalization of the extremely dry water level in the lake regions caused low-elevation depressions and mudflats to evolve into grassy islets, forming new breeding habitats for snails and even resulting in increased snail density [46]. The global warming and redistribution of precipitation caused by climate change aggravate the complexity of the spatio-temporal evolution and growth and decline of the snail population in the above area [30, 33].

The number of sites with an RR value > 1 for an interaction effect between 2015 and 2019 (90 [2015], 70 [2016], 65 [2017], 86 [2018] and 81 [2019]) showed a V-shaped trend of “falling first and then rising” (Fig. 5). This may be related to the serious flooding in the Yangtze River basin in 2016. Serious flooding events result in snails being submerged underwater for a long time, which is not conducive to the survival of adult snails [19]. It is possible that snails migrate passively in floodwaters and spread to the snail-free surroundings, forming emerging habitats [6, 41]. Therefore, the density of snails generally decreased briefly in the year following the flood, then rebounded due to compensation by new young snails [45, 47].

### Implications of the findings on snail control

Attention should be paid to the fluctuation and slight increase in snail density reported in this study. Environmental changes (such as those due to floods and water conservancy projects) may contribute to the diffusion of snail habitats and the rebound of snail density [41, 45, 47]. The Yangtze River Economic Belt prohibits large-scale use of molluscicides and environmental modification measures [24, 42]. This requires us to strengthen precise control and develop new environmentally friendly molluscicides. The findings of this study will help clarify and identify snail-infested settings. Low night light, suitable climate and rich vegetation will increase snail density in the downstream area of the TGD; consequently, priority in snail investigations should be on areas with those characteristics. In the upstream area, more surveillance resources should be concentrated on sites with abundant rainfall, flat slopes and lush vegetation.

### Strengths and deficiencies of the study

This study had two major strengths. First, we demonstrated the difference in influencing factors between the upstream and downstream areas of the TGD based on a 5-year longitudinal study. Second, we analyzed the spatial–temporal effects on the change in snail density between the 12th year and the 17th year after the TGD became operational. To the best of our knowledge, few similar studies have previously been published. There are still some limitations that should be mentioned. First, factors were estimated by Kriging interpolation or extracted from remote sensing that might result in a measurement bias. Second, this study did not incorporate flooding duration and snail control measures due to a lack of such data.

## Conclusions

Collectively, our findings suggested an obvious fluctuation in snail density in the downstream area and a slightly rising trend in snail density in the upstream area. This trend of change may be related to flooding disasters and the Yangtze River Protection Program. Snail density demonstrated a rebound between 2015 and 2019. In particular, temperature, humidity, vegetation and human activity were the main drivers affecting the snail abundance in the downstream area of the TGD, while precipitation, slope and vegetation were the main drivers affecting upstream snail abundance. The differential determinants are associated with different geography and socio-economic factors of two schistosomiasis endemic areas. These findings can assist authorities to develop and perform more precise strategies for surveys and control of snail populations.

## Supplementary Information

**Additional file 1: Table S1.** Parameter selection of five Bayesian models. **Table S2.** Correlation analysis of variables. **Table S3.** The diagnosis of collinearity. **Table S4.** Model selection.

## Data Availability

Supporting data for the conclusions of this article are included within the article. The raw data supporting the conclusions of this article will be made available upon reasonable request.

## Abbreviations

CAR: Conditional autoregressive process
CI: Confidence interval
DIC: Deviance information criterion
DW: Distance to major waterway
GPS: Global positioning system
GST: Average annual ground surface temperature
LAMP: Loop-mediated isothermal amplification
NDVI: Normalized Difference Vegetation Index
NL: Annual nighttime light index
Pre: Total precipitation
RH: Average annual relative humidity
RR: Relative risk
SLP: Slope
SSH: Sunshine hour
Tem: Annual average temperature
TGD: Three Gorges Dam
Tmax: Average maximum temperature in July
Tmin: Average minimum temperature in January
VIF: Variance inflation factor
