# Supplementary material for: Three Gorges Dam: Potential differential drivers and trend in the spatio-temporal evolution of the change in snail density based on a Bayesian spatial–temporal model and 5-year longitudinal study
Source: Parasit Vectors. 2023 Jul 14;16:232. doi: 10.1186/s13071-023-05846-6 (PMC10349508; doi:10.1186/s13071-023-05846-6)
Supplement: Supplementary file 1 — Additional file 1: Table S1. Parameter selection of five Bayesian models. Table S2. Correlation analysis of variables. Table S3. The diagnosis of collinearity. Table S4. Model selection. [file 13071_2023_5846_MOESM1_ESM.docx]

**Supplementary materials**

Table S1 Parameter selection of five Bayesian models

| Model | Name | Formula |
| --- | --- | --- |
| Model1 | Non-spatiotemporal model | $log\left( u_{it} \right)=\beta_{0}+\sum\beta_{k}X_{itk}$ |
| Model2 | Temporal independent model | $log\left( u_{it} \right)=\beta_{0}+\sum\beta_{k}X_{itk}+\varphi_{t}+\gamma_{t}$ |
| Model3 | Spatial independent model | $log\left( u_{it} \right)=\beta_{0}+\sum\beta_{k}X_{itk}+u_{i}+v_{i}$ |
| Model4 | Spatiotemporal independence model | $log\left( u_{it} \right)=\beta_{0}+\sum\beta_{k}X_{itk}+u_{i}+v_{i}+\varphi_{t}+\gamma_{t}$ |
| Model5 | Spatiotemporal interaction model | $log\left( u_{it} \right)=\beta_{0}+\sum\beta_{k}X_{itk}+u_{i}+v_{i}+\varphi_{t}+\gamma_{t}+\delta_{it}$ |

Table S2 Correlation analysis of variables

| Variables | Snail density | Tem | Tmin | Tmax | Pre | RH | SSH | GST | DW | NDVI | SLP | NL |
| --- | --- | --- | --- | --- | --- | --- | --- | --- | --- | --- | --- | --- |
| Snail density | 1.00 |  |  |  |  |  |  |  |  |  |  |  |
| Tem | -0.08* | 1.00 |  |  |  |  |  |  |  |  |  |  |
| Tmin | 0.04 | 0.03 | 1.00 |  |  |  |  |  |  |  |  |  |
| Tmax | 0.01 | 0.42* | -0.35* | 1.00 |  |  |  |  |  |  |  |  |
| Pre | 0.11* | -0.05 | -0.06 | 0.36* | 1.00 |  |  |  |  |  |  |  |
| RH | 0.15* | 0.04 | -0.27* | 0.48* | 0.39* | 1.00 |  |  |  |  |  |  |
| SSH | -0.08* | 0.99* | 0.03 | 0.42* | -0.04 | 0.04 | 1.00 |  |  |  |  |  |
| GST | -0.08* | 0.91* | 0.11* | 0.31* | -0.13* | -0.15* | 0.91* | 1.00 |  |  |  |  |
| DW | 0.05 | -0.01 | 0.01 | 0.06 | 0.06 | 0.12* | -0.01 | -0.01 | 1.00 |  |  |  |
| NDVI | 0.16* | -0.08* | 0.17* | -0.10* | 0.02 | -0.01 | -0.08* | -0.06 | 0.26* | 1.00 |  |  |
| SLP | 0.03 | -0.14* | 0.34* | -0.42* | -0.07* | -0.34* | -0.14* | -0.03 | 0.07* | 0.29* | 1.00 |  |
| NL | -0.11* | -0.02 | -0.16* | -0.01 | -0.09* | -0.07* | -0.02 | -0.04 | -0.18* | -0.47* | -0.14* | 1.00 |

Tem annual average temperature; Tmin average minimum temperature in Jan; Tmax average maximum temperature in Jul; Pre total precipitation; RH average annual relative humidity; SSH sunshine hour; GST average annual ground surface temperature; DW distance to major waterway; NDVI normalized difference vegetation index; SLP slope; NL annual night-time light index

* The correlation between two variables was statistically significant at *P* < 0.05

Table S3 The diagnosis of collinearity

| Variables | Tem | Tmin | Tmax | Pre | RH | SSH | GST | DW | NDVI | SLP | NL |
| --- | --- | --- | --- | --- | --- | --- | --- | --- | --- | --- | --- |
| VIF | 7087.54 | 1.39 | 2.24 | 1.60 | 1.78 | 7022.41 | 7.82 | 1.10 | 1.45 | 1.46 | 1.33 |

Tem annual average temperature; Tmin average minimum temperature in Jan; Tmax average maximum temperature in Jul; Pre total precipitation; RH average annual relative humidity; SSH sunshine hour; GST average annual ground surface temperature; DW distance to major waterway; NDVI normalized difference vegetation index; SLP slope; NL annual night-time light index; VIF, variance expansion factor

Table S4 Model selection

| Model | DIC |
| --- | --- |
| Model1 | 1302341.18 |
| Model2 | 1273343.18 |
| Model3 | 451557.64 |
| Model4 | 574174.05 |
| Model5 | 8202.02 |

DIC deviance information criterion
